# Supplementary figures and images for: Interactive effects of acacia biochar, maize hybrids, and irrigation levels on soil health and crop productivity
Source: PeerJ. 2025 Sep 24;13:e20048. doi: 10.7717/peerj.20048 (PMC12476171; doi:10.7717/peerj.20048)

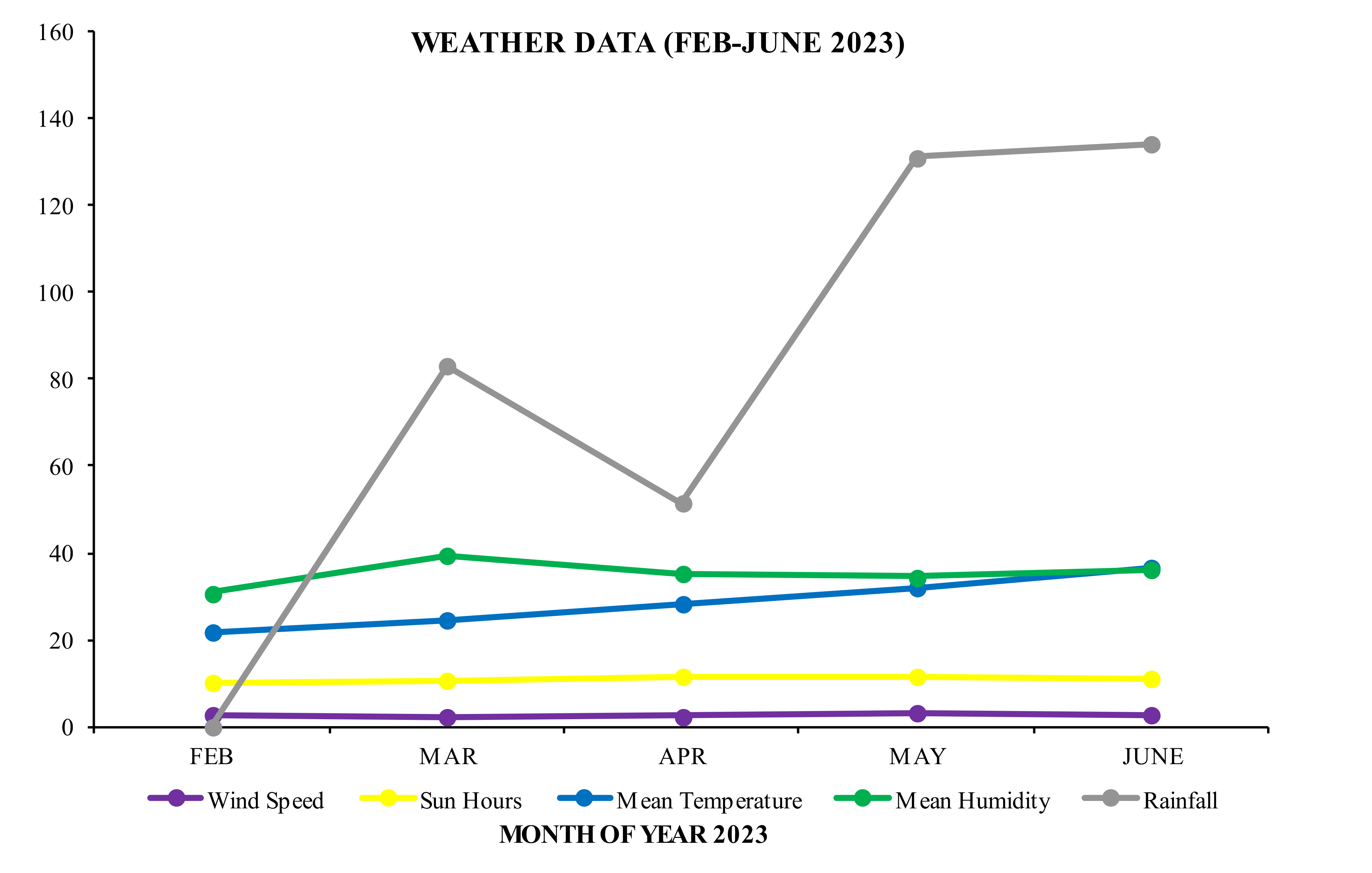

Supplement: Supplemental Information 2 [file peerj-13-20048-s002.png]

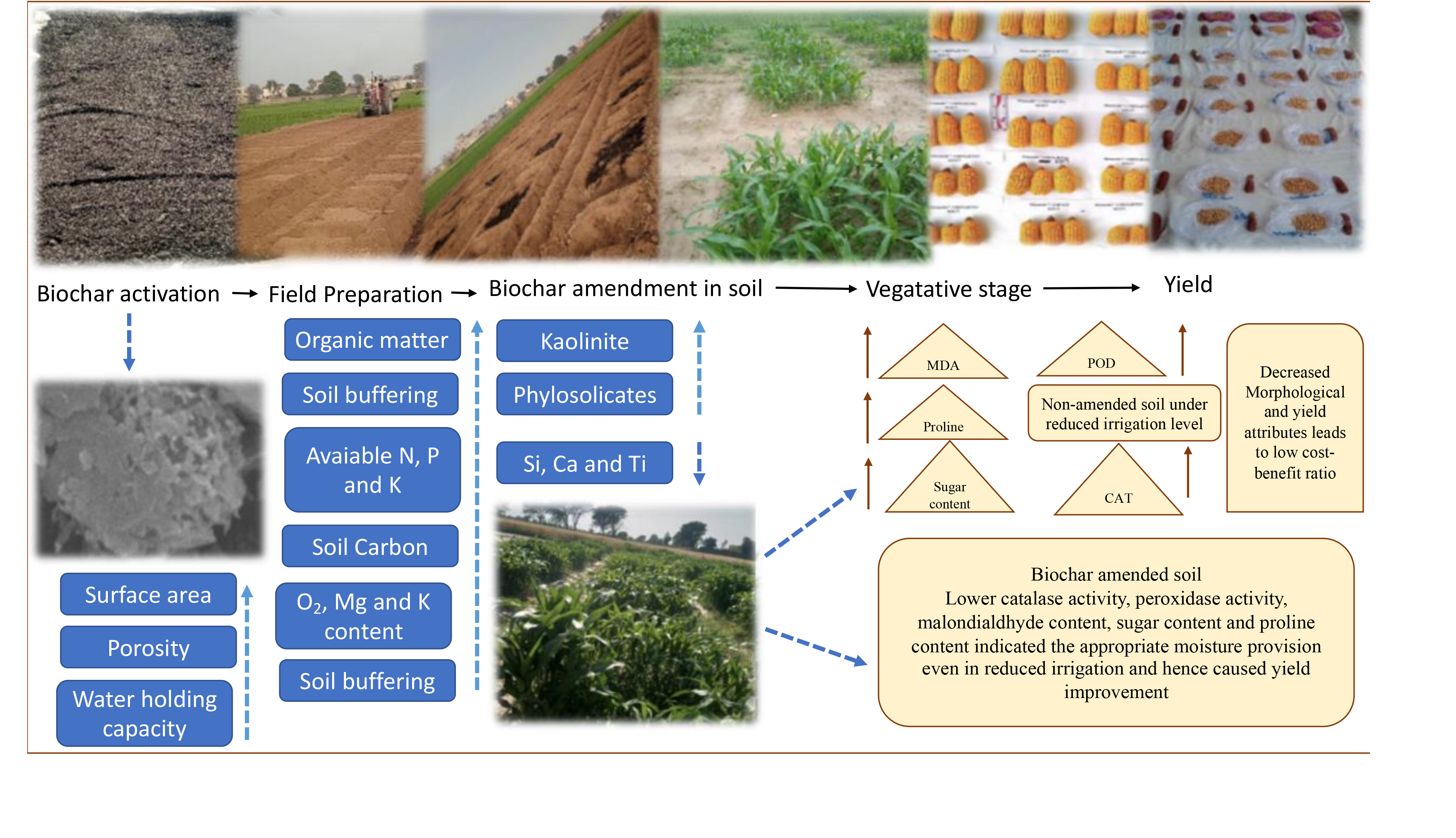

Supplement: Supplemental Information 8 [file peerj-13-20048-s008.png]
