# Supplementary material for: Interactive effects of acacia biochar, maize hybrids, and irrigation levels on soil health and crop productivity
Source: PeerJ. 2025 Sep 24;13:e20048. doi: 10.7717/peerj.20048 (PMC12476171; doi:10.7717/peerj.20048)
Supplement: Supplemental Information 3 — Different lowercase letter assessed by 3-way ANOVA of biochar rates, irrigation levels and maize hybrids indicates level of significance at 95% confidence interval. Means sharing different letters have significant differences at P < 0.05%. 0 tons ha−1 (A0), 5 tons ha−1 (A1), 10 tons ha−1 (A2) of activated biochar. [file peerj-13-20048-s003.txt]

| **Table 2** Impact of activated biochar amendment in soil on three maize hybrids shoot fresh weight (SFW), shoot dry weight (SDW) under FI, PDI, and SDI. | | | | | | |
| --- | --- | --- | --- | --- | --- | --- |
| **Treat** | **Vegetative** | | **Tasseling** | | **Maturity** | |
|  | **SFW** | **SDW** | **SFW** | **SDW** | **SFW** | **SDW** |
| V1A0F1 | 292.5±0.82 h | 12.9±0.33 ef | 452.2±2.06 g | 203.6±0.28 d | 835.88±2.34 m | 334.3±0.93 h |
| V1A0PDI | 206.2±0.475 n | 6.85±0.22 jk | 437.3±1.92 i-k | 174.1±0.40 j | 825±1.9 n | 280.5±0.64 k |
| V1A0SDI | 148.6±0.16 t | 3.3±0.18 m | 422.6±0.70 mn | 157± 0.61 o | 782.7±085 q | 234.8±0.25 t |
| V1A1FI | 301.8±0.56 f | 19.9±0.17 c | 468.1±1.41 b-e | 250±0.90 c | 862.4±1.60 j | 344.9±064 f |
| V1A1PDI | 213.3±0.39 m | 12.6±0.13 de | 445.7±3.65 g-i | 181±0.23 h | 853.2±1.57 k | 298±0.53 k |
| V1A1SDI | 165.2±0.77 r | 4.50±2.67 lm | 434.9±1.76 jl | 161±0.59 n | 826±3.88 n | 247.8±1.16 r |
| V1A2F1 | 312.4±0.52 c | 23.6±0.08 b | 480±2.15 ce | 260 ±0.26 c | 892.7±1.49 c | 357±0.59 c |
| V1A2PDI | 220.6±0.26 j | 14.6±0.28 de | 453.6±0.85 fg | 195±0.62 e | 882.4±1.05 de | 300±0.35 j |
| V1A2SDI | 172.5±0.29 q | 6.25±0.19 jl | 444.6±0.57 km | 187±0.22o | 862.8± 1.48 ij | 258.8±0.44 p |
| V2A0F1 | 293.7±0.46 gh | 15.2±0.22 d | 453.2±1.12 fg | 174± 0.38 j | 839.2±1.33 lm | 336.6±0.53 gh |
| V2A0PDI | 204.9±0.22 n | 10.5±2.09 gh | 427.7±2.10 lm | 171± 0.24 k | 825±0.90 n | 278.2±0.30 n |
| V2A0SDI | 161.6±0.33 s | 5.03±0.11 k-m | 415.2±3.55 n | 154±0.90 p | 808.1±1.68 o | 269.2±0.50 r |
| V2A1FI | 304.3±0.25 e | 20.1±0.38 c | 468.8±0.95 b-e | 223±0.48 a | 869.6±0.73 gh | 336.6±0.53 gh |
| V2A1PDI | 214.5±0.38 m | 16.7±0.12 d | 475.1±2.81 bd | 185±1.02 g | 858.2±1.53 jk | 328.2±0.30 n |
| V2A1SDI | 188.5±0.36 o | 7.02±0.11 k-m | 434.2±2.45 j-l | 161±0.37 n | 842.8±1.83 q | 342.2±0.50 r |
| V2A2F1 | 314.5±0.38 b | 25±0.35 b | 488.3±2.1bc | 245±0.75 a | 898.8±1.1 c | 377.8±0.29 e |
| V2A2PDI | 218.4±0.67 k | 19.9±0.12 c | 481.7±0.85 b | 195±0.22 e | 873.8±2.68 fg | 361.7±0.52 m |
| V2A2SDI | 172.6± 0.45 q | 12.1±0.16 fg | 448.4±0.57 a | 164±0.24 m | 863±2.28 ij | 397.8±0.5 s |
| V3A0FI | 294.9±0.84 g | 15.8±0.35 d | 466.9±1.26 de | 210±0.29 c | 842.8±2.40 l | 337.1±0.68 g |
| V3A0PDI | 205.5± 0.36 n | 9.2±0.15 hi | 463.4±2.10 e | 190±0.36 f | 822.2±1.45 n | 279.5±0.96 n |
| V3A0SDI | 160.8±0.40 s | 4.94±0.12 k-m | 438.4±3.55 i-k | 171±0.31 k | 804.2±2.04 o | 241.9±0.49 r |
| V3A1FI | 306.9±0.50 d | 23±0.38 b | 476.6±1.62 bc | 214±0.28 b | 877±1.45 ef | 350.8±0.61 d |
| V3A1PDI | 216.7±0.37 l | 13.9±0.22 ef | 470.7±3.55 ef | 194.7±0.32 g | 857.1±1.51 hi | 331.8±0.58 m |
| V3A1SDI | 222.5±0.43 a | 9.85±0.22 jk | 462.8±1.62 hj | 179.2±0.37 m | 829.2±1.57 a | 327.9±0.5 n |
| V3A2FI | 350.1± 0.46 e | 30.8±0.52 a | 494.3±3.55 a | 222.5±0.43 a | 920±1.73 b | 397.6±0.62 a |
| V3A2PDI | 281.9±0.91 q | 16.9±0.33 ef | 488±1.12 a | 210± 0.46 e | 906.1±1.51 d | 388.3±0.58 i |
| V3A2SDI | 254.5±0.82 | 12.9±0.34 ij | 477±0.70 b | 207.2±0.28 I | 895±2.28 c | 375.8±0.45 o |
| Different lowercase letter assessed by 3-way ANOVA of biochar rates, irrigation levels and maize hybrids indicates level of significance at 95% confidence interval. Means sharing different letters have significant differences at *P* < 0.05%.  0 tons ha^−1^ (A0), 5 tons ha^−1^ (A1), 10 tons ha^−1^ (A2) of activated biochar.  Full irrigation (FI), partially deficit irrigation (PDI), and severely deficit irrigation (SDI). | | | | | | |
