# Supplementary material for: Interactive effects of acacia biochar, maize hybrids, and irrigation levels on soil health and crop productivity
Source: PeerJ. 2025 Sep 24;13:e20048. doi: 10.7717/peerj.20048 (PMC12476171; doi:10.7717/peerj.20048)
Supplement: Supplemental Information 4 — Different lowercase letter assessed by 3-way ANOVA of biochar rates, irrigation levels and maize hybrids indicates level of significance at 95% confidence interval. Means sharing different letters have significant differences at P < 0.05%. 0 tons ha−1 (A0), 5 tons ha−11 (A1), 10 tons ha−1 (A2) of activated biochar Full irrigation (FI), partially deficit irrigation (PDI), and severely deficit irrigation (SDI) [file peerj-13-20048-s004.docx]

| **Table 3** Impact of activated biochar amendment in soil on three maize hybrids root fresh weight (RFW), root dry weight (RDW) under FI, PDI, and SDI. | | | | | | |
| --- | --- | --- | --- | --- | --- | --- |
| **Treat** | **Vegetative** | | **Tasseling** | | **Maturity** | |
|  | **RFW** | **RDW** | **RFW** | **RDW** | **RFW** | **RDW** |
| V1A0F1 | 16.4±1.07 h-k | 5.23±0.07 i-m | 43.6±2.31 h | 12.2±0.16 g | 64.4±2.20 h | 17.9±0.42 d-h |
| V1A0PDI | 10.4±0.58 mn | 2.67±0.05 n-q | 28.1±1.64 kl | 6.15±0.27 k | 44.4±2.20 j | 10.7±0.31 g-j |
| V1A0SDI | 7.0±1.00 n | 1.15±0.07 q | 20.0±1.01 m | 4.21±0.11 m | 34.4±1.74 k | 6.68±0.19 j |
| V1A1FI | 19.7±0.85 f-h | 9.2±0.02 de | 53.3±2.17 ef | 15.9±0.13 e | 87.8±1.40 bc | 25.8±0.32 b-d |
| V1A1PDI | 14.5±0.95 j-l | 6.71±0.03 f-i | 48±1.31 f-h | 12.6±0.12 g | 77.7±1.79 ef | 20.1±0.02 c-e |
| V1A1SDI | 11.6±0.83 lm | 3.21±0.09 m-q | 24.2±1.06 lm | 4.76±0.19 lm | 43.4±1.77 j | 8.67±0.01 i-j |
| V1A2F1 | 27±1.80 e | 11.8±0.05 bc | 67.3±2.16 b | 18.9±0.07 c | 107.1±1.53 a | 30.03± 0.28 b |
| V1A2PDI | 22.6±1.98 f | 9.5±0.02 de | 59.7±1.60 d | 16.7±0.28 d | 82±1.79 ef | 22.8±0.13 b-e |
| V1A2SDI | 15.5±0.81 i-k | 6.40±0.01 f-i | 31.1±1.03 jk | 6.25±0.15 k | 48.5±1.0 j | 9.70± 0.03 h-j |
| V2A0F1 | 16.8±1.05 h-j | 4.51±0.04 j-n | 43.9±2.31 h | 12.1±0.08 g | 69.1±1.22 h | 16.4±0.15 e-i |
| V2A0PDI | 12.9±0.84 k-m | 3.25±0.03 o-q | 37.1±2.30 ij | 9.3±0.11 i | 45.5±1.63 j | 11.3±0.14 g-j |
| V2A0SDI | 9.8±0.63 mn | 3.0±0.05 pq | 25.5±1.36 k-m | 5.03±0.30 l | 33.4±2.06 k | 6.64±0.10 j |
| V2A1FI | 26.8±1.07 e | 7.71±0.05 e-h | 57.6±2.06 de | 16.1±0.12 de | 87.8±2.35 bc | 19.2±0.11 d-g |
| V2A1PDI | 19.3±1.22 f-h | 6.64±0.02 g-j | 50.6±1.55 fg | 12.7±0.16 g | 74.2±1.41 fg | 18.5±0.14 d-h |
| V2A1SDI | 15.4±0.99 i-k | 4.1±0.01 bc | 34.4±1.07 ij | 6.74±0.28 jk | 48.2±1.03 j | 9.62±0.01 h-j |
| V2A2F1 | 35.4±1.44 c | 10.1±0.02 cd | 70.5±1.29 b | 20.1±0.28 b | 102.7±2.20 a | 28.8±0.15 bc |
| V2A2PDI | 26.8±2.28 e | 7.81±0.01 e-h | 66.1±1.35 bc | 16.7±0.26 d | 89.2±1.42 b | 21.8±0.13 b-e |
| V2A2SDI | 19.0±0.35 f-h | 5.77±0.05 h-l | 60.8±1.44 cd | 11.1±0.02 h | 56.6±0.84 i | 11.1±0.50 g-i |
| V3A0FI | 18.4±0.93 g-i | 4.63±0.02 l-p | 46.3±2.09 gh | 12.6±0.28 g | 67.1±1.32 h | 18.8±0.21 d-j |
| V3A0PDI | 12.9±1.02 k-m | 3.43±0.01 q | 36.4±2.06 ij | 9.2±0.15 i | 44.4±2.66 j | 11.8±0.028 f-h |
| V3A0SDI | 10.6±0.23 mn | 2.85±0.02 df | 24.2±1.53 lm | 3.94±0.12 l | 33.4±2.13 k | 6.32±0.15 j |
| V3A1FI | 40.3±1.13 b | 14.85±0.06 h-k | 65.8±2.00 bc | 18.4±0.31 c | 92.8±1.32 b | 27.9±0.05 bc |
| V3A1PDI | 28.3±1.06 de | 9.4±0.02 k-o | 59.4±1.53 d | 14.6±0.22 f | 86.4±0.86 cd | 22.8±0.098 b-e |
| V3A1SDI | 21.9±1.53 fg | 4.88±0.01 a | 37.3±2.32 i | 7.29±0.34 j | 47.4±1.52 j | 6.71±0.14 j |
| V3A2FI | 46±1.49 a | 12.4±0.02 b | 78.2±2.00 a | 21.4±0.42 a | 106.5±1.87 a | 49.3±0.50 a |
| V3A2PDI | 31.5±0.78 d | 10.92±0.04 f-I | 67.8±1.87 b | 16.5±0.40 de | 92.2±2.10 b | 29.9±0.16 bc |
| V3A2SDI | 26.7±0.94 e | 7.83±0.03 df | 60.5±1.99 ef | 12.2±0.21 h | 85.2±1.25 i | 10.8±0.29 d-h |
| Different lowercase letter assessed by 3-way ANOVA of biochar rates, irrigation levels and maize hybrids indicates level of significance at 95% confidence interval. Means sharing different letters have significant differences at *P* < 0.05%.  0 tons ha^−1^ (A0), 5 tons ha^−1^ (A1), 10 tons ha^−1^ (A2) of activated biochar  Full irrigation (FI), partially deficit irrigation (PDI), and severely deficit irrigation (SDI) | | | | | | |
