# Supplementary material for: Interactive effects of acacia biochar, maize hybrids, and irrigation levels on soil health and crop productivity
Source: PeerJ. 2025 Sep 24;13:e20048. doi: 10.7717/peerj.20048 (PMC12476171; doi:10.7717/peerj.20048)
Supplement: Supplemental Information 5 — Different lowercase letter assessed by 3-way ANOVA of biochar rates, irrigation levels and maize hybrids indicates level of significance at 95% confidence interval. Means sharing different letters have significant differences at P < 0.05%. 0 tons ha−1 (A0), 5 tons ha−1 (A1), 10 tons ha−1 (A2) of activated biochar Full irrigation (FI), partially deficit irrigation (PDI), and severely deficit irrigation (SDI) [file peerj-13-20048-s005.docx]

| **Table 4** Impact of activated biochar amendment in soil on three maize hybrids leaf fresh weight (LFW), leaf dry weight (LDW) under FI, PDI, and SDI at vegetative, tasseling, and maturity stage. | | | | | | |
| --- | --- | --- | --- | --- | --- | --- |
| **Treat** | **Vegetative** | | **Tasseling** | | **Maturity** | |
|  | **LFW** | **LDW** | **LFW** | **LDW** | **LFW** | **LDW** |
| V1A0F1 | 29.9±0.32 h-j | 6.52±0.15 c | 87.1±1.01 c | 74.8±0.24 h | 133.5±0.78 gh | 37.8±1.6 e |
| V1A0PDI | 23.2±1.91 lm | 3.65±0.55 de | 73.4±2.44 de | 43.3±0.18 no | 114.8±1.46 k | 26.7±2.61 i-k |
| V1A0SDI | 17.1±1.46 n | 2.3±0.1 f-i | 54.7±1.32 g | 30.5±0.219 t | 85.3±1.35 n | 12.8±0.69 n |
| V1A1FI | 41.3±1.31 c-e | 8.57±0.11b | 98.9±1.38 b | 79.5±0.42 g | 144.8±0.92 f | 42.8±1.45 d |
| V1A1PDI | 32.1±0.91 g-i | 4.40±0.42 d | 84.3±1.75 c | 49.9±0.26 p | 123.3±1.03 ij | 30.6±1.09 f-i |
| V1A1SDI | 21.2±1.5 mn | 2.69±0.35 e-i | 66.8±1.99 f | 33.6±0.27 s | 103.5±0.80 l | 21.4±1.18 lm |
| V1A2F1 | 43.6±2.16 b-d | 9.91±0.05 a | 116.5±0.88 a | 89.1±0.82 e | 167.3±1.54 d | 52.5±2.39 b |
| V1A2PDI | 35.4±1.81 f-g | 4.43±0.24 d | 94.7±0.99 b | 54.9±0.22 m | 144.8±2.56 f | 44.2±0.90 cd |
| V1A2SDI | 33.8±1.02 f-h | 2.63±0.41 e-i | 77.8±1.78 d | 41.9±0.30 p | 106.7±1.57 l | 28.5±1.39 g-j |
| V2A0F1 | 41.9±1.68 c-e | 4.58±0.21 d | 84.9±2.12 c | 65.1±0.23 j | 130.9±1.86 h | 31.9±1.54 f-h |
| V2A0PDI | 27.8±1.94 i-l | 3.0±0.13 e-h | 57.3±1.63 g | 36.4±0.44 s | 124.3±3.01 i | 26.5±1.59 i-k |
| V2A0SDI | 20.1±1.02 mn | 2.16±0.21 hi | 46.3±2.67 h | 27.5±0.52 q | 93.9±2.92 m | 13.5±1.50 n |
| V2A1FI | 45.2±1.06 bc | 9.18±0.32 ab | 93.9±1.61 b | 99.3±0.25 d | 176.9±2.12 c | 48.0±1.84 c |
| V2A1PDI | 37.0±1.54 ef | 4.4±0.97 d | 65.5±1.10 f | 44.6±0.41 n | 137.1±0.99 g | 30.6±1.51 f-i |
| V2A1SDI | 23.4±1.21 km | 2.25±0.17 g-i | 53.4±0.83 g | 39±1.04 c | 118.7±0.53 jk | 21.7±1.31 lm |
| V2A2F1 | 52.8±1.57 a | 10.1±0.06 a | 117.9±1.50 a | 88.6±0.41 e | 193.4±1.58 a | 52.5±1.51 b |
| V2A2PDI | 45.2±1.21 bc | 6.59±0.31 c | 87.7±1.95 c | 75.7±1.40 m | 173.9±2.97 c | 44.2±1.34 cd |
| V2A2SDI | 33.8±2.05 f-h | 5.46±0.02 d-g | 65.5±1.73 f | 55.7±2.40 m | 123.3±2.44 ij | 28.5±0.66 g-j |
| V3A0FI | 43.2±1.02 b-d | 6.93±1.01 c | 87.1±1.01 c | 58.7±0.40 l | 151.3±2.27 e | 34.5±0.95 ef |
| V3A0PDI | 28.4±0.71 ij | 3.65±0.39 de | 73.4±2.44 de | 34.2±0.43 r | 143±1.40 f | 23.1±1.02 kl |
| V3A0SDI | 21.6±1.14 mn | 2.48±0.38 i | 54.7±1.32 g | 26.3±0.20 t | 83.3±2.30 n | 12.8±1.5 n |
| V3A1FI | 47.6±1.34 b | 9.31±0.49 ab | 98.9±1.38 b | 123.3±0.39 b | 184.2±0.97 b | 46±1.95 cd |
| V3A1PDI | 37±2.89 ef | 5.93±0.23 c | 84.3±1.75 c | 75.6±0.67 h | 175.5±1.42 c | 36.4±1.51 e |
| V3A1SDI | 26.7±0.91 jk | 2.58±0.55 e-i | 66.8±1.99 f | 59.6±0.15 kl | 95.8±1.27 m | 18.8±1.56 m |
| V3A2FI | 55.4±1.01 a | 9.79±0.96 a | 116.5±0.88 a | 137.6±0.30 a | 192.7±0.8 a | 55.1±1.1 ab |
| V3A2PDI | 36±3.20 fg | 6.28±0.73 c | 94.7±0.99 b | 84.5±0.80 f | 184.2±2.55 b | 43.1±0.98 d |
| V3A2SDI | 28.7±1.02 ij | 3.49±0.08 d-f | 68.5±1.42 ef | 65.5±0.42 j | 114.1±1.62 k | 25.1±0.99 j-l |
| Different lowercase letter assessed by 3-way ANOVA of biochar rates, irrigation levels and maize hybrids indicates level of significance at 95% confidence interval. Means sharing different letters have significant differences at *P* < 0.05%.  0 tons ha^−1^ (A0), 5 tons ha^−1^ (A1), 10 tons ha^−1^ (A2) of activated biochar  Full irrigation (FI), partially deficit irrigation (PDI), and severely deficit irrigation (SDI) | | | | | | |
