# Supplementary material for: Interactive effects of acacia biochar, maize hybrids, and irrigation levels on soil health and crop productivity
Source: PeerJ. 2025 Sep 24;13:e20048. doi: 10.7717/peerj.20048 (PMC12476171; doi:10.7717/peerj.20048)
Supplement: Supplemental Information 6 — Different lowercase letter assessed by 3-way ANOVA of biochar rates, irrigation levels and maize hybrids indicates level of significance at 95% confidence interval. Means sharing different letters have significant differences at P < 0.05%. 0 tons ha−1 (A0), 5 tons ha−1 (A1), 10 tons ha−1 (A2) of activated biochar Full irrigation (FI), partially deficit irrigation (PDI), and severely deficit irrigation (SDI) [file peerj-13-20048-s006.docx]

| **Table 5** Impact of activated biochar amendment in soil on three maize hybrids plant height, leaf area at vegetative, tasseling, and maturity stage and grain fresh weight and grain dry weight at maturity stage under FI, PDI, and SDI. | | | | | | | | |
| --- | --- | --- | --- | --- | --- | --- | --- | --- |
| **Treatment** | **Plant height (cm)** | | | **Leaf area (cm)** | | | **Grain fresh Wt (g)** | **Grain dry Wt (g)** |
|  | **Vegetative stage** | **Tasseling stage** | **Maturity** | **Vegetative stage** | **Tasseling stage** | **Maturity** | **Maturity** | **Maturity** |
| V1A0F1 | 96.4±1.09 mn | 253.5±1.11 ij | 298.5±1.45 b | 131.5±1.77 s | 367.7±11.7 1j | 877.9± 1.6 fg | 51.63± 0.98 o | 29.1±0.11 i |
| V1A0PDI | 76.4±2.64 0 | 242.2±1.02 kl | 283.1±1.04 jk | 124.2±0.77 t | 336.1± 2.06 lm | 853.4±1.06 hi | 44.02±1.56 p | 14.2±1.20 lm |
| V1A0SDI | 54.9±1.27 p | 216.0±2.86 q | 248.4±1.77 n | 116.3±1.05 u | 316.9±2.91 m | 802.8±1.85 mn | 16.28± 0.92 s | 6.23±0.55 m |
| V1A1FI | 125.4±1.03 gh | 277.4±2.15 ef | 307.9±1.24 h | 165.5±0.88 q | 407.7±1.65 h | 884.2±1.07 ij | 74.60±1.82 kl | 34.1±0.78 g |
| V1A1PDI | 109.5±1.43 k | 266.5±2.74 gh | 286.5±2.03 j | 146.3±0.88 r | 397.9±2.06 h | 860.1±0.91h | 54.13± 1.0 o | 25.6±1.59 jk |
| V1A1SDI | 93.2±1.35 mn | 234.2±1.28 l-n | 257.5±0.95 m | 134.3±2.0 s | 376.6±1.48 ij | 824.8±0.91k | 35.87±1.83 q | 13.3±0.66 m |
| V1A2F1 | 186.3±1.12 a | 296.5±2.14 bc | 340.3±1.93 c | 178.2±0.95 o | 427.7±1.56 fg | 898.0±0.99 d | 118.60± 0.95 gh | 48.5±1.29 e |
| V1A2PDI | 112.4±1.52 jk | 279.5±1.66 de | 327.1±0.73 e | 172.8±1.53 p | 412.7± 0.99 gh | 872.8±1.55g | 67.7±1.55 mn | 35.3±1.27 g |
| V1A2SDI | 97.8±1.72 lm | 245.5±2.52 jk | 321.3±0.86 fg | 168.1±1.57 pq | 402±0.9 h | 844.7± 1.03 ij | 76.7±0.99 k | 28.3±1.42 ij |
| V2A0F1 | 116.6±1.21 ij | 232.6±2.33 m-o | 318.4±1.81g | 271.8±1.00 l | 394.1±1.64 hi | 819.2±0.97 kl | 82.4±0.58 j | 33.2±0.69 gh |
| V2A0PDI | 102.3±2.08 l | 224.1±1.30 o-q | 251.7± 1.04 n | 268.2±1.29 l | 372.7± 1.96 j | 802.3±0.98 mn | 68.6±0.61 mn | 17.2±1.53 l |
| V2A0SDI | 92.3±0.77 n | 218.3±0.88 p-q | 248.2± 1.04 n | 254.6±1.38 i | 361.0± 0.99 jk | 797.5±1.47 n | 43.3±1.045 p | 11.1±1.0 m |
| V2A1FI | 126.2±2.03gh | 244.6±2.29 jk | 327.8±1.91e | 296.9±1.20 i | 462.3± 0.85 d | 874.6±1.28 fg | 133.2±1.063 d | 77.8±1.59 c |
| V2A1PDI | 120.3±0.86 i | 237.4±0.43 k-m | 278.7±2.86 kl | 286.9±2.03 j | 442.5± 0.68 ef | 826.9±2.68 k | 118.4±1.22 gh | 48.5±1.28 e |
| V2A1SDI | 93.2±1.61 mn | 227.1±2.35 n-p | 275.4±2.39 l | 276.5±2.10 k | 429.3± 0.86 fg | 816.9±1.93 kl | 64.8±1.74 n | 22.7±0.80 k |
| V2A2F1 | 141.3±1.11 cd | 298.5±0.97 b | 346.9±1.67 b | 318.5± 0.9 f | 551.3±1.01 a | 921.5±1.71 b | 125.5±1.27 ef | 84.4±1.05 b |
| V2A2PDI | 137.1±2.26 de | 270±2.34 fg | 339.9±1.67 c | 311.3± 0.94 g | 527.7±1.53 b | 893.1±1.67 de | 94.2±0.96 i | 52.6±2.21 d |
| V2A2SDI | 127.5±3.07 fg | 257.6±1.1 hi | 282.1±1.04 jk | 302.6±1.78 h | 518.6±1.41 b | 883.8± 0.98 ef | 78.5±0.83 jk | 28.4±1.01 ij |
| V3A0F1 | 121.5±1.81 hi | 252.6±0.97ij | 324.1±1.84 ef | 293.0±1.93 i | 457.1±1.53 de | 837.8±1.21 j | 96.6±2.55 i | 30.6±1.68 hi |
| V3A0PDI | 116.7±3.23 ij | 241.7±0.92 k-m | 320.6±0.78 fg | 262.7±1.16 m | 367.5±1.11 j | 819.6± 0.77kl | 71.0± 0.95 lm | 28.1±1.05 ij |
| V3A0SDI | 109.7±1.11 k | 238.4 ±0.82 k-m | 294.7±1.15 i | 256.4±1.24 n | 344.5±1.27 kl | 810.4±1.11lm | 28.4±1.39 r | 13.3±0.59 m |
| V3A1F1 | 142.8± 2.04 c | 287.9±1.29 cd | 342.6±1.42 bc | 346.7±1.52 c | 489.5±1.24 c | 908.7±1.13 c | 152.2±1.27 c | 55.6±1.02 d |
| V3A1PDI | 127.4±2.07 fg | 273.89±1.95 g | 332.4±1.05 d | 336.4±1.32 d | 442.6± 0.93 ef | 892.5± 0.98 de | 129.1±1.04 de | 44.9±10.6 f |
| V3A1SDI | 118.0±2.09 i | 266.6±1.32 gh | 324.6±1.40 ef | 327.8±1.91e | 440.3±1.03 ef | 872.8±1.22 g | 113.8± 2.08 h | 22.6±1.07 k |
| V3A2F1 | 151.1±0.85 b | 308.3±1.16 a | 352.7±0.93 a | 362.1±1.42 a | 570.2± 0.69 a | 933.9±1.654 a | 198.5±1.05 a | 87.9±1.24 a |
| V3A2PDI | 145.4±2.49 c | 295.2±1.27 bc | 346.4±1.03 b | 357.4±1.56 b | 531.2±2.07 ab | 922.7±1.35 b | 173.5±0.76 b | 47.9±1.57 ef |
| V3A2SDI | 132.4±1.03 ef | 290.6±1.20 bc | 309.7±0.90 h | 350±0.97 c | 443.1±0.89 b | 898±1.85 d | 124.1±2.3 f | 25.3±1.26 jk |
| Different lowercase letter assessed by 3-way ANOVA of biochar rates, irrigation levels and maize hybrids indicates level of significance at 95% confidence interval. Means sharing different letters have significant differences at *P* < 0.05%.  0 tons ha −1 (A0), 5 tons ha −1 (A1), 10 tons ha −1 (A2) of activated biochar  Full irrigation (FI), partially deficit irrigation (PDI), and severely deficit irrigation (SDI) | | | | | | | | |
