# Supplementary material for: Interactive effects of acacia biochar, maize hybrids, and irrigation levels on soil health and crop productivity
Source: PeerJ. 2025 Sep 24;13:e20048. doi: 10.7717/peerj.20048 (PMC12476171; doi:10.7717/peerj.20048)
Supplement: Supplemental Information 7 [file peerj-13-20048-s007.docx]

| Table S1 Average economic gain percentage of three maize hybrids DK-2088 (V1), Yh-5427 (V2), DK-6317 (V3) grown under full irrigation (FI), partially deficit irrigation (PDI), and severely deficit irrigation (SDI) in 0 tons ha^−1^ (A0), 5 tons ha^−1^ (A1), and 10 tons ha^−1^ (A2) activated biochar amendment in soil | | | | | | | | | | | |
| --- | --- | --- | --- | --- | --- | --- | --- | --- | --- | --- | --- |
| DK-2088 | Yield kg ha^−1^ | Net profit USD ha^−1^ | Percent increase over control | Yh-5427 | Yield kg ha^−1^ | Net profit USD ha^−1^ | Percent increase over control | DK-6317 | Yield kg ha^−1^ | Net profit USD ha^−1^ | Percent increase over control |
| V1A0F1 | 6890 c-f | 1109.4 | 164 | V2A0F1 | 6556.25 d-f | 1056 | 155 | V3A0F1 | 6468.13 e-g | 902.9 | 133 |
| V1A0PDI | 5343.75 ik | 647.3 | 95 | V2A0PDI | 5192.71 i-l | 594.87 | 87 | V3A0PDI | 5337.50 i-k | 623 | 91 |
| V1A0SDI | 4291.67 m | 238 | 35 | V2A0SDI | 4081.04 m | 199 | 29 | V3A0SDI | 4076.04 m | 184.2 | 27 |
| V1A1FI | 7252.1 b-d | 1303.4 | 192 | V2A1FI | 7397.71 bc | 1323 | 195 | V3A1FI | 8309.38 a | 1552 | 228 |
| V1A1PDI | 5784.79 g-i | 796.88 | 117 | V2A1PDI | 6150.62 f-h | 908.3 | 134 | V3A1PDI | 6727.08 b-d | 1080 | 159 |
| V1A1SDI | 4751.9 j-m | 463.17 | 68 | V2A1SDI | 4751.9 j-m | 402 | 59 | V3A1SDI | 4494.38 l-m | 215.4 | 31 |
| V1A2F1 | 7886.46 ab | 1526 | 224 | V2A2F1 | 8344.79 a | 1628 | 239 | V3A2F1 | 8608.54 a | 1771.2 | 261 |
| V1A2PDI | 6617.71 d-f | 1087.3 | 160 | V2A2PDI | 6617.7 d-f | 1065 | 156 | V3A2PDI | 7156.25 b-e | 1259 | 185 |
| V1A2SDI | 5537.5 hi | 637 | 94 | V2A2SDI | 4628.8 j-m | 603 | 88 | V3A2SDI | 5359.37 i-j | 642 | 94 |
| Different lowercase letter assessed by 3-way ANOVA of biochar rates, irrigation levels and maize hybrids indicates level of significance at 95% confidence interval. Means sharing different letters have significant differences at *P* < 0.05%. | | | | | | | | | | | |
